# Antiviral Peptides (AVPs) of Marine Origin as Propitious Therapeutic Drug Candidates for the Treatment of Human Viruses

**DOI:** 10.3390/molecules27092619

**Published:** 2022-04-19

**Authors:** Linda Sukmarini

**Affiliations:** Research Center for Applied Microbiology, National Research and Innovation Agency (BRIN), Jl. Raya Bogor Km. 46, Cibinong 16911, West Java, Indonesia; linda.sukmarini@brin.go.id

**Keywords:** antiviral peptides, infectious diseases, marine peptides, natural products, therapeutic drugs

## Abstract

The marine environment presents a favorable avenue for potential therapeutic agents as a reservoir of new bioactive natural products. Due to their numerous potential pharmacological effects, marine-derived natural products—particularly marine peptides—have gained considerable attention. These peptides have shown a broad spectrum of biological functions, such as antimicrobial, antiviral, cytotoxic, immunomodulatory, and analgesic effects. The emergence of new virus strains and viral resistance leads to continuing efforts to develop more effective antiviral drugs. Interestingly, antimicrobial peptides (AMPs) that possess antiviral properties and are alternatively regarded as antiviral peptides (AVPs) demonstrate vast potential as alternative peptide-based drug candidates available for viral infection treatments. Hence, AVPs obtained from various marine organisms have been evaluated. This brief review features recent updates of marine-derived AVPs from 2011 to 2021. Moreover, the biosynthesis of this class of compounds and their possible mechanisms of action are also discussed. Selected peptides from various marine organisms possessing antiviral activities against important human viruses—such as human immunodeficiency viruses, herpes simplex viruses, influenza viruses, hepatitis C virus, and coronaviruses—are highlighted herein.

## 1. Introduction

Infectious diseases, mainly caused by viral pathogens, remain a primary global health issue that has already caused a high mortality rate [1]. Many of the most threatening or deadliest human infectious diseases are caused by viruses, such as human immunodeficiency viruses (HIVs) [2,3], influenza viruses [4,5], hepatitis viruses [3,6], and the recent emerging pandemic threat of severe acute respiratory syndrome coronavirus 2 (SARS-CoV-2) [7,8]. In spite of striking development in vaccines and small-molecule antiviral drugs, new emerging and re-emerging viral disease outbreaks and the progression of antiviral drug resistance and drug toxicity have forced scientists to perpetually probe novel antiviral agents. Moreover, only a few therapeutic drugs are available for various viruses. Due to their excellent efficacy, high selectivity, and low potential for resistance development, the development of peptide-derived drugs to overcome their constraints on stability and bioavailability is becoming a point of interest in the pharmaceutical industry [9,10,11]. Hence, antiviral peptides (AVPs) that mainly originate from antimicrobial peptides (AMPs) with antiviral activities can be prospective antiviral agents to fight viral infections.

AVPs are typically short peptides (generally consisting of 12–50 amino acid residues) with positively charged (typically +2 to +9) and amphipathic structures [12,13,14,15,16,17]. In addition to these general features enabling these peptides to serve as antimicrobials (including antibacterials, antifungals, and antiparasitics), hydrophobicity is likely to be a key characteristic for AVPs to target enveloped viruses [18,19]. It is worth mentioning that previous statistical-analysis-based studies on the Antimicrobial Peptide Database (APD) have shown that hydrophobic cysteine residues are abundant in AVPs [20,21].

Interestingly, as a part of the immune system in all living organisms, these promiscuous peptides are the first line of defense against various pathogens, including viruses. Naturally occurring AMPs with antiviral properties have been found in almost all multicellular organisms, ranging from plants, animals, mammals, and microbes to marine entities. Marine organisms are highly regarded reservoirs of pharmacologically active molecules, including peptides. These natural-product-based peptides evolve naturally through structural modification to adapt to a harsh marine environment. The adapted features eventually enable them to sustain biological properties against pathogens; thus, marine-based peptides have been continuously considered as potential anti-infective drug candidates [22,23].

Many of the reviews reported previously on such topics include anti-infective activities of marine peptides, various antiviral compounds from marine organisms, and peptide-derived antiviral drugs [14,24,25,26,27,28,29]. Moreover, reports on in silico computer-based AVP screenings and the compilation of databases covering experimentally tested peptides for use against clinically significant viruses have also been described elsewhere [30,31,32,33]. However, this short review focuses on recent marine-peptide-based antiviral compounds. It presents thorough coverage of AVPs—either naturally occurring peptides or synthetic ones—derived from marine species such as microbes, invertebrates, and vertebrates over the past decade (2011–2021). This review aims to summarize those selected natural-product-derived peptides demonstrating considerable antiviral activity in vitro and/or in vivo, or even under clinical study against human viruses such as HIVs, influenza viruses, herpes simplex viruses (HSVs), hepatitis C virus (HCV), or SARS-CoV-2. These peptides are currently isolated from marine organisms or chemically synthesized, or are previously known compounds that have been recently reported. In addition, the biosynthetic origin of AVPs and their antiviral modes of action are also featured herein.

## 2. Biosynthesis of AVPs: A Brief Overview

Biosynthetically generated through ribosomal or non-ribosomal machinery, the vast chemical structures of peptides from natural sources—including AVPs—can differ from linear to cyclic, incorporating canonical and non-canonical amino acids. Essentially, the ribosomal peptides, also designated as ribosomally synthesized and post-translationally modified peptides (RiPPs), are built upon a set of only 20 standard canonical amino acid residues, while non-ribosomal peptides (NRPs) can also contain a larger pool of building blocks of both canonical and non-canonical amino acids [34,35].

The chemical diversity of RiPPs is generated by the extensive post-translational modifications (PTMs) whereby their precursor peptides are modified by dedicated modifying enzymes encoded in the biosynthetic gene cluster. In brief, the modified peptides subsequently undergo proteolytic cleavage of the leader peptide sequences in the precursor peptides, and additional PTMs of further modified peptides can be present in certain conditions. Thus, this leads to the export of mature and active peptides from the cells [35,36].

On the other hand, being decoupled from the ribosome, NRPs are synthesized by a multifunctional modular enzyme complex, namely, non-ribosomal peptide synthetases (NRPSs). This machinery assembly line generally consists of initiation, elongation, and termination modules. Each module comprises at least three catalytic domains: (1) an adenylation domain to select a specific amino acid monomer, (2) a thiolation domain to covalently bind the activated monomer, and (3) a condensation domain to catalyze peptide elongation. The product release of NRPS assembly termination can be linear, cyclic, or cyclodepsipeptides. Moreover, further extensive explanations of the NRPs’ synthesis have been reviewed so far [36,37,38].

## 3. Antiviral Mechanism of Action of AVPs

**Figure 1:**
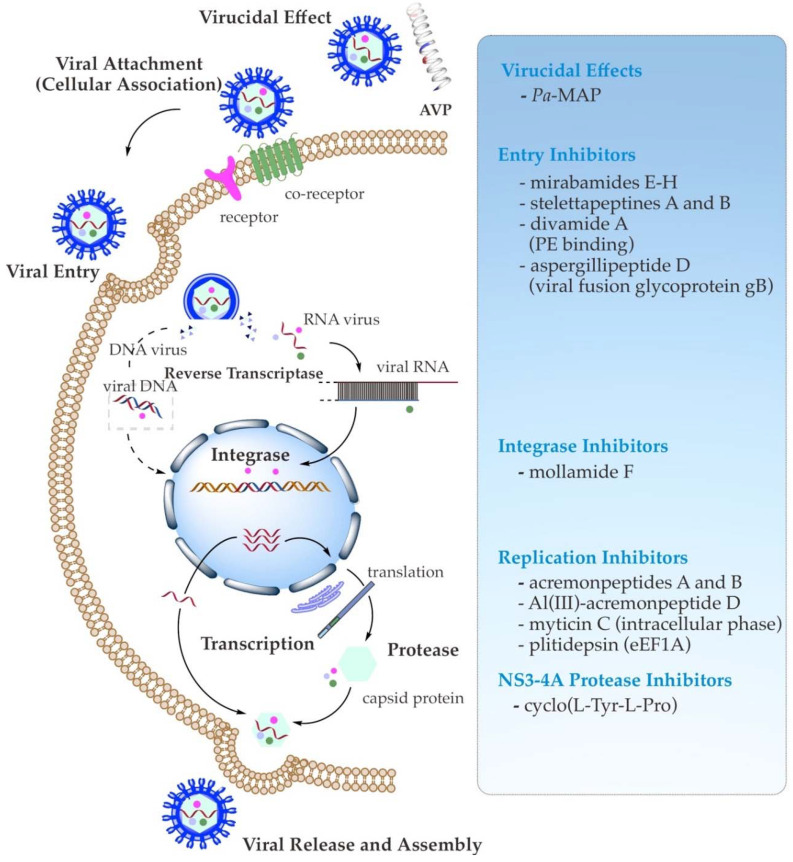
Schematic summary of known antiviral mechanisms of the recently reported marine AVPs.

As specific antiviral drugs are mainly dedicated to treating particular viruses, different phases of the viral life cycle have been used to search for novel antiviral drugs. AVPs act against enveloped viruses by interrupting the fundamental stages of their life cycle of entry, synthesis, or assembly. Their inhibition sites include the viral particle or virion inhibition (virucidal effect), adsorption (cellular association), viral penetration, endosomal escape, viral uncoating, viral genome replication, and viral assembly, packaging, and release [14,39]. Moreover, the proposed mechanism of action of antiviral activity of AVPs may generally encompass (1) their direct binding to the viral target, which is involved in the direct inhibition of host cell infection or viral pathogenesis; (2) their attachment to the target on the host surface (indirect inhibition), which is engaged in the competition or interaction with functional surface proteins of viruses; and (3) their indirect virus-targeting function through suppression of the viral gene expression, as well as inhibition of the viral enzymes, e.g., viral polymerase and integrase, related to the intracellular replication and transcription (biological function inhibition) (Figure 1) [29,39,40,41,42].

### 3.1. Direct Binding Inhibition (Virucidal Effect)

The binding to host cells through interaction with functional receptors is the initial trend in viral infection. Once the viral particle attaches to a host cell, its genetic material is inserted into the cell during this initial attachment and penetration stage. Regarding direct virion inhibition, the AVP papuamide A has shown an immediate virucidal effect of HIV-1 inhibition through a viral-membrane-targeting mechanism [43,44]. This marine peptide bears a tyrosine hydroxy and an aliphatic moiety tail that have been suggested to interact with the cholesterol membrane of the virus target and assist with insertion into the viral membrane, respectively, resulting in the subsequent viral membrane disruption and, ultimately, viral inactivation [44].

### 3.2. Viral Attachment (Cellular Association) and Entry Inhibition

The indirect binding, as evidenced by the HIV-1-targeting α-defensin human neutrophil peptide HNP-1, has been found to bind viral envelope Env and host CD4 glycoproteins/co-receptors in a glycan- and serum-independent manner. In addition, the oligomerization or refolding of HNP-1 could block the viral fusion. Hence, this peptide requires a multifaceted mode of action for HIV entry inhibition [45,46].

Moreover, several studies [41,47,48,49,50] have reported that two mechanisms of the cellular association or viral attachment may prevent the entry of the enveloped viruses. In the instance of the activity towards the influenza virus, firstly, the AVP competes with sialic acid to bind with the envelope glycoprotein—namely, hemagglutinin (HA)—and clogs its receptor site, inhibiting the influenza virion from interacting with the host cell membrane. The peptide can imitate sialic acid’s behavior to be recognized by the receptor-binding site of viral HA. Furthermore, the binding of AVP to the other main component of the influenza virus—namely, *N*-acetylneuraminic acid—on the host cell surface can also prevent viral attachment to host cells. Secondly, the conformation of HA is inhibited; thus, intracellular entry is prohibited, leading to endosomal escape and viral genome release [41]. A similar antiviral activity targeting primary attachment has also been found to inhibit HSV and hepatitis viruses. The AVP with a positive charge and good hydrophobicity binds to a cellular glycan moiety to prevent HSV from attaching to the host cell surface [51]. This glycan moiety is heparan sulfate, which is known to be a negatively charged glycosaminoglycan that favors HSV viral particles through its basic positively charged binding pocket in the virion glycoprotein to attach to the host cell surface. Therefore, the AVP can prevent HSV virion invasion by binding to glycosaminoglycan molecules as a receptor, hampering the interaction of the virus and the receptor in a host cell. With respect to hepatitis viruses—particularly HCV—some AVPs have also been demonstrated to interact with the virion receptors and co-receptors. AVPs that resemble the cellular protein apolipoprotein E (ApoE) can break up the glycan-dependent interaction or attachment of HCV, hindering entry and infection of target host cells (hepatocytes) [52,53]. As observed by Chi et al. (2016) [54], the HCV fusion-inhibitory peptide could block viral envelope glycoproteins’ E1/E2-mediated membrane fusion by interfering with E1 and E2 heterodimerization. Moreover, the peptide was likely to provoke the dimer E1/E2 glycoproteins’ conformational changes, impairing HCV membrane fusion. Additionally, another AVP (CL58) seemed to inhibit viral entry, possibly after initial binding (post-binding) of the co-receptor cellular membrane protein CD81, and just prior to the final intracellular fusion in endosomes [55,56]. Furthermore, these mentioned inhibition mechanisms of the viral membrane fusion stage by peptides have been further comprehensively discussed elsewhere [57].

### 3.3. Viral Enzymes and Replication Inhibition

In addition, AVPs have also been reported to inhibit the viral replication of HSV-2, influenza viruses, and HCV. In the case of HSV-2, the AVPs can block the transport of a primary viral protein named VP16 into the nucleus. This transcriptional protein regulator induces immediate expression of viral genes required for survival at the initial cellular response. Some AVPs that act on influenza viruses also target viral-RNA-dependent RNA polymerase, comprising PB1, PB2, and PA subunits. These subunits control polymerization and endonuclease cleavage, recognition, binding to the host mRNAs, and endonuclease activity towards host pre-mRNA. Binding those subunits, AVPs can also prohibit the assembly of the influenza polymerase complex [13,41,58]. For HCV, AVPs can act on NS3-4A—a multifunctional enzyme with serine proteinase and helicase functions that are harnessed for HCV replication [59,60]. Furthermore, to inhibit the replication of the very recent virus SARS-CoV-2, Tonk et al. (2021) [61] and Heydari et al. (2021) [62] gathered findings on a number of antiviral activities of AVPs that could be involved. Some AVPs directly act on the viral envelope (virucidal effect), binding to the viral spike glycoprotein that blocks the interaction with angiotensin-converting enzyme-2 (ACE2) in host cells, hampering endosomal acidification for uncoating throughout the initial viral life cycle, or escorting the host ACE2 receptor [63,64,65,66,67].

Moreover, several approaches have been deployed to evaluate the antiviral activity of marine peptides, including neutralization, viral titration, cell viability, and virus plaque-reduction assays [27]. Some selected examples of marine AVPs that possess antiviral activity against important human enveloped viruses—such as HIV, influenza, HSV, HCV, and even SARS-CoV-2—are summarized in Figure 1, listed in Table 1, and featured below.

## 4. Anti-HIV (Human Immunodeficiency Virus) Marine AVPs

**Figure 2:**
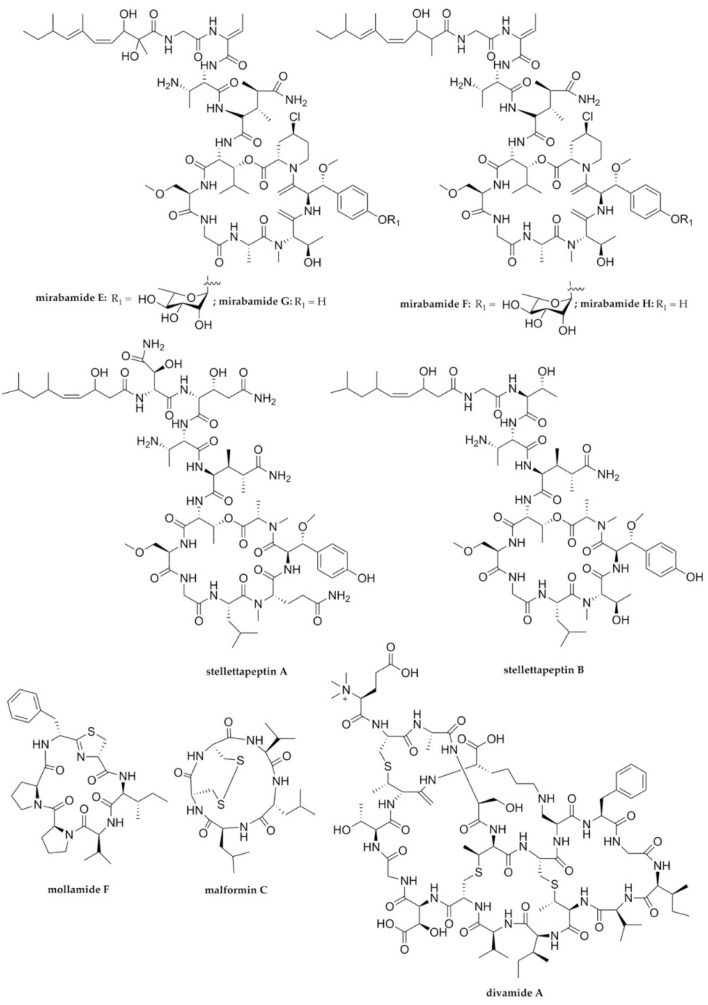
The chemical structures of marine-derived peptides with anti-HIV activity.

Various commercially available anti-HIV drugs pose several restrictions, including toxicity, resistance, side effects, and long-term treatment, which have limited the application of these drugs. They may break down at a subsequent phase due to severe adverse effects and the emergence and spread of drug-resistant strains [25,81]. However, the development of one of the marine-derived anti-HIV compounds entering clinical evaluation discloses the excellent potential of marine natural products, underlining the continuous prerequisite of progress in anti-HIV drugs derived from living entities. The compound is a lectin-based griffithsin originally obtained from a red alga [82,83]. Many more potential anti-HIV therapeutics from marine resources have also been previously described [84,85]. Moreover, recently reported AVPs derived from marine-sourced organisms have demonstrated anti-HIV activity, including mirabamides E–H, stellettapeptines A and B, mollamide F, malformin C, and divamide A (Figure 2 and Table 1).

The exploration of marine sponges as a prosperous source of antiviral lead structures [86] has led to the isolation of new *Stelletta*-derived mirabamides E–H (Figure 2). Along with the known mirabamide C, these cyclic depsipeptides (cyclodepsipeptides) have been obtained from the sponge *Stelletta clavosa* Ridley collected from the Torres Strait, Queensland, Australia. In addition, the extraction of mirabamides from other phylogenetically different sponges associated with diverse microbes—such as *Siliquariasspongia mirabilis*—suggests that mirabamides are possibly derived from microbes or bacteria [68]. Structurally, the new cyclodepsipeptides mirabamides E–H are likely to be previously recognized peptide-based HIV-1 entry inhibitors—the mirabamides A–D and papuamides [87]. Moreover, based on a neutralization assay using pseudotyped viruses with an enveloped HIV-1 strain and tested with the known mirabamide C, these new cyclodepsipeptides exhibit considerable antiviral replication activity towards the genital epithelial cells expressing CD4 and CCR5 HIV-1 co-receptors (IC_50_ = 121, 62, 68, and 41 nM, for mirabamides E–H, respectively). Hence, all four new depsipeptides seem to be potent inhibitors of HIV-1 at the entry stage of infection. Apparently, this class of mirabamides binds to the HIV-1 envelope glycoprotein to inhibit viral fusion into the host cell membrane [87]. However, mirabamides E–H differ in having an unprecedented 2-amino-2-butenoic acid moiety instead of threonine. It is noteworthy that the twofold elevation of the activity of mirabamides F and G compared with mirabamide C was likely due to the residue alteration. A similar change in the hydrophobicity of 2,3-dihydroxy-2,6,8-trimethyldeca-(4*Z*,6*E*)-dienoic acid (Dhtda) with 3-hydroxy-2,6,8-trimethyldeca-(4*Z*,6*E*)-dienoic acid (Htda) yielded a twofold increase in the inhibitory effect (observed with mirabamide E compared to mirabamide F, as well as with mirabamide G compared to mirabamide H). These results imply that the hydrophobicity is attributable to the potency of inhibition to some extent [68].

The other recent sponge genus *Stelletta*-derived anti-HIV cyclodepsipeptides—stellettapeptines A and B (Figure 2)—were successfully isolated from a *Stelletta* sp. obtained from northwestern Australia [69]. Stellettapeptines bear a 3-hydroxy-6,8-dimethylnon-4-(*Z*)-enoic acid—a unique non-canonical amino acid moiety of the polyketide subunit first identified in these cyclodepsipeptides. In addition, unprecedented 3-hydroxyglutamine and 3-hydroxyasparagine residues have also been found in these peptides. Similar to mirabamides, stellettapeptines are suggested to be derived from bacterial origin, as they possess the characteristic of non-ribosomal peptides or non-ribosomal peptide–polyketide hybrids, and are phylogenetically discrete from sponge-derived callipeptines and papuamides. Both of these new cyclic peptides were found to be remarkably active against HIV-1. Their antiviral activities have been assessed in an XTT-based cell viability assay employing HIV-1_RF_-infected CEM-SS human T cells. Stellettapeptines A and B strongly inhibited HIV-1-infection-induced cytopathy, associated with apoptosis (EC_50_ = 23 and 27 nM, respectively) [69]. The composition and cyclic configuration of non-canonical amino acids is likely of great importance for antiviral activity [88]. Therefore, similar to the cyclodepsipeptides mentioned above—papuamides and mirabamides—stellettapeptines may also target the viral membrane and act on viral entry. However, the further mechanism of viral entry inhibition of stellettapeptines has not been well described.

In a conventional bioactivity-guided screening campaign of antiviral drug discovery targeting HIV by an international cooperative biodiversity group (ICBB) between the United States and Papua New Guinea, two new thiazoline cyclopeptides—mollamides E and F—have been discovered. Together with a new Tris-phenethyl urea called molleurea A, they were isolated from the extract of a tunicate *Didemnum molle* collected from Papua New Guinea [70]. It should be pointed out that those cyclopeptides were first recognized by metagenome sequencing of the symbiont cyanobacterium *Prochloron didemni*, whereby their predicted products were detected from a single colony of the ascidian, or by an *Escherichia coli* heterologous expression system of the engineered corresponding ribosomal biosynthetic route [89]. Nevertheless, Lu et al. (2012) [70,90] reported that this finding constituted the first time these peptides had been fully depicted as isolated marine natural-product-based compounds. Moreover, the configuration of these peptides employing the advanced Marfey’s approach showed that the thiazoline and phenylalanine in both mollamides E and F were in d-configuration instead of in the natural l-configuration of ribosomal peptides. Mollamide F showed HIV inhibition with an IC_50_ value of 78 µM based on a cytoprotective assay, and an IC_50_ value of 39 µM based on an HIV integrase inhibition assay, while no activity was observed in mollamide E in either assay. It has been previously reported that dolastatin 3, which is structurally similar to mollamides, exhibits HIV integrase activity [91]. HIV-1 integrase possessed by the virus is an enzyme vital for integrating the HIV genome or genetic material into the host genome within the cell, and is therefore regarded as an interesting target for therapeutic anti-HIV agents [92]. This would thus suggest that mollamide F could indirectly act on the viral target by inhibiting the biological function of the viral protein in terms of enzymatic activity of integrase.

In addition to promising bioactive peptides from invertebrates and their bacterial symbionts as mentioned above, marine endophytic fungi have also been a source of propitious antiviral lead compounds [93,94]. One of the more potent anti-HIV agents has been recently reported from a known mycotoxin compound—malformin C [71]. In addition to its original antibacterial activity [95,96], this fungal cyclic pentapeptide has also previously been found to bestow antiparasitic [97] and cytotoxic activities [98,99]. The rediscovered malformin C as an anti-HIV agent was extracted from the fermentation broth of the marine-derived black aspergilli *Aspergillus niger* SCSIO Jscw6F30. Along with new aspernigrins and the other known compounds rubrofusarins and fonsecin, Zhou et al. (2016) [71] isolated malformin C from a marine alga *Sargassum* sp. obtained from the island of Yongxiang in the South China Sea. This recent marine fungal cyclopentapeptide displayed vigorous inhibitory activity towards the chemokine receptor CCRC5 in tropic HIV-1 SF162 infection in TMZ-bl cells, with an IC_50_ value of 1.4 μM. Interestingly, this significant anti-HIV activity was equivalent to that of a nucleoside reverse transcriptase inhibitor (abacavir; IC_50_ value of about 0.8 μM) and an effective HIV-entry inhibitor (ADS-JI; IC_50_ value of about 1.8 μM). However, the mechanism of action of its antiviral function has not yet been reported.

Current advancements engaged in (meta)genomics and synthetic biology have also led to another breakthrough finding of excellent anti-HIV peptides from the genus *Didemnum* [72,100]. Initially, the biological-activity-assay-guided fractionation of the tunicate *D. molle* E11-036 showed remarkable anti-HIV activity, unlike those from neighboring colony extracts of E11-037. The active compound was identified as divamide A (Figure 2)—a ribosomal lanthipeptide containing *N*-trimethylglutamate—and the partial precursor peptide sequence motif “GTTR.” To further decipher structural confirmation, due to insufficient material, the compound was synthesized by the symbiotic cyanobacteria *Prochloron didemni* living in the marine tunicate *Didemnum molle* E11-036 through the expression of a *div* biosynthetic gene cluster. The gene cluster was reconstituted and expressed in *Escherichia coli*. Moreover, the generated semi-synthetic divamide A displayed significant HIV inhibition activity towards CEM^TART^-T cells, with an IC_50_ value of 0.225 µM [72]. Likewise, other lanthipeptides, such as cinnamycin and duramycin, show high binding affinity and specificity to phosphatidylethanolamine (PE); divamide A presumably interacts with this viral envelope lipid, thereby blocking viral entry [101]. PE is abundant in enveloped viruses, including HIV-1, in which it is derived from the budding process at the plasma membrane of infected cells. [102,103,104,105]. Due to its extensive distribution in many enveloped viruses, viral lipid PE-targeted AVPs such as lanthipeptides possess a wide spectrum of activity against various human pathogenic viruses, including Ebola, Dengue, West Nile, and Zika viruses—exemplified by duramycins [106,107]—and HSV-1, as presented by cinnamycin [107,108,109].

## 5. Anti-Influenza-Virus Marine AVPs

**Figure 3:**
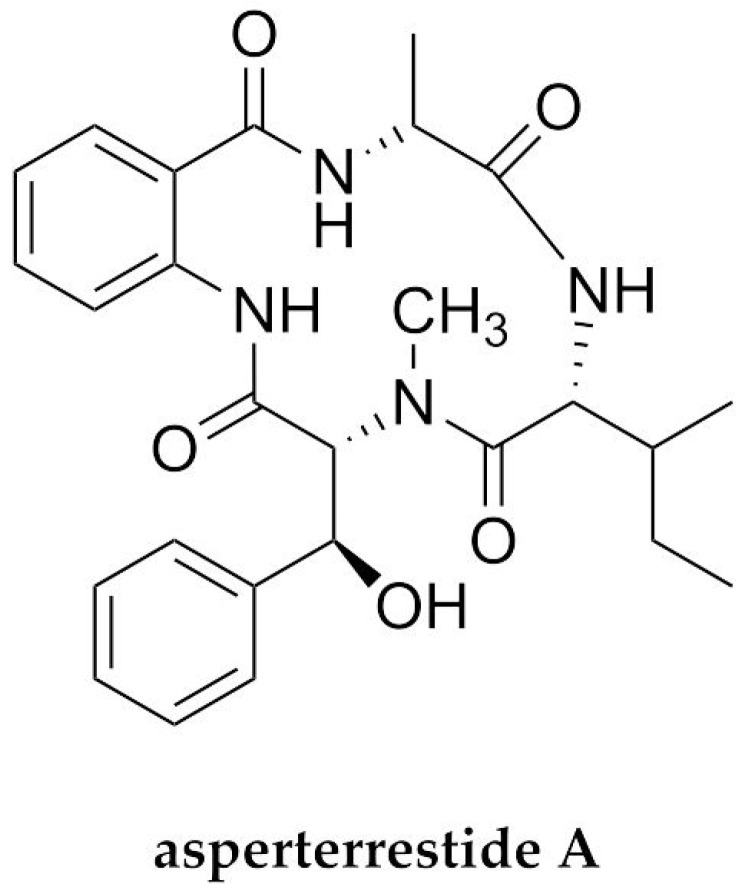
The chemical structure of asperterrestide A—a marine-derived fungal peptide possessing inhibitory effects on influenza viruses.

One of the emerging and drug-resistant infectious diseases that has received significant attention is pneumonia or respiratory infections caused by influenza viruses. The most notable feature of influenza viruses is their expeditious evolution, generating extensive variability of strains—particularly the influenza A virus. There have been several outbreaks of influenza viruses, including H1N1, or Spanish flu (1918); H2N2, or Asian flu (1957); H3N2, or Hong Kong flu (1968); and the recent generation of influenza viruses in South-East Asia (A/H5N1; 1998) and the Netherlands and Belgium (A/H7N7; 2003). The many antiviral drugs used for influenza treatment include neuraminidase inhibitors (e.g., zanamivir and oseltamivir) as well as adamantane drugs (e.g., rimantadine and amantadine), which are not able to minimize the risk of adverse effects [25]. Moreover, one of the recent promising AVPs with inhibitory effects against influenza viruses but less toxicity is marine-fungus-derived asperterrestide A [73] (Figure 3 and Table 1).

Asperterrestide A—a cyclotetrapeptide with a rare 3-hydroxy-*N*-methyl-Phe moiety—was obtained from the cultivation broth of the marine gorgonian *Echinogorgia aurantiaca*-derived fungus *A. terreus* SCSGAF1062. In addition to its cytotoxicity against cancer cells, asperterrestide A has demonstrated inhibitory effects on an M2-resistant influenza virus strain A/W/33 (H1N1) and an M2-sensitive strain A/Hong Kong/8/68 (H3N2), with IC_50_ values of 20.2 and 0.41 µM, respectively, based on a cytopathic assay. It is likely that the potential of its antiviral activity was due to the presence of the rare moiety mentioned above [73]. However, this is a rather insufficient understanding of the antiviral mode of action of this fungal-derived AVP.

## 6. Anti-HSV (Human Simplex Virus) Marine AVPs

**Figure 4:**
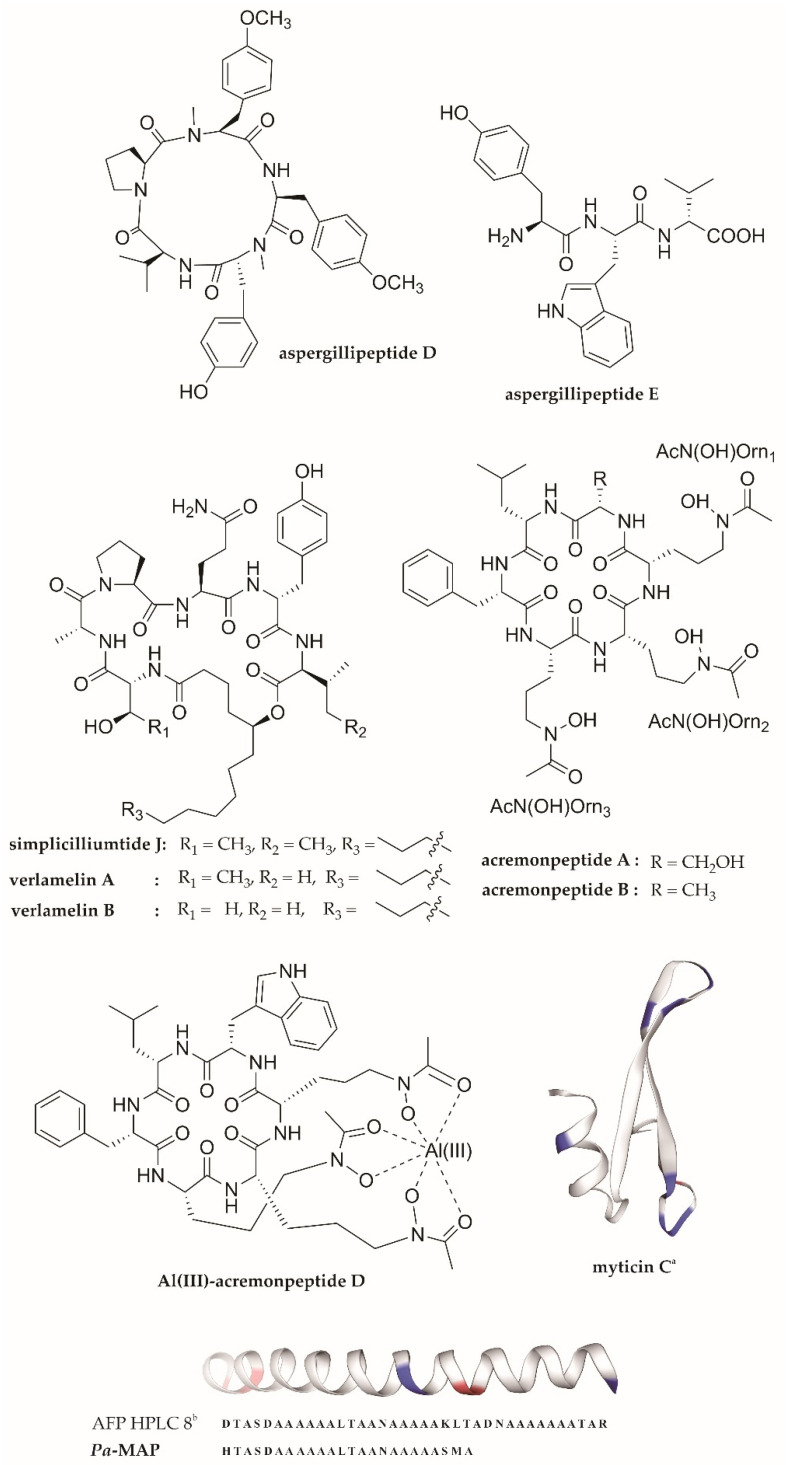
The structures of marine peptides demonstrating anti-HSV activity: ^a^ The structure of myticin C was generated using SWISS-MODEL modelling (https://swissmodel.expasy.org, accessed on 23 February 2022) [110,111], based on the amino acid sequence from GenBank with accession number AEZ79080.1 [112]. Moreover, the synthetic *Pa*-MAP is derived from the antifreeze peptide (AFP) HPLC-8^b^ (as shown by the amino acid sequence), the structural model of which was retrieved from the SWISS-MODEL repository (https://swissmodel.expasy.org/repository, accessed on 23 February 2022) [113], with the UniProtKB AC number Q99013 (ANPB_ PSEAM) [114]. The blue and red colors within the structural model indicate positively and negatively charged residues, respectively.

The first marine-derived compound with antiviral activity that reached the market was vidarabine (Ara A). This sponge-derived nucleoside compound was licensed and approved as an anti-HSV drug [86]. Since it was later found to be less efficient than the newer commercial drug Zovirax (acyclovir), vidarabine was discontinued. However, due to viral resistance to acyclovir, more effective new antiviral drugs with high selectivity remain to be developed—especially those from peptide-based marine AVPs. These include marine fungal peptides, marine invertebrate peptides, and marine vertebrate peptides, which have all been found to demonstrate anti-HSV activities (Figure 4 and Table 1).

In addition to the potent anti-HIV peptide malformin C and anti-influenza peptide asperterrestide A, a number of recently isolated and reported marine-derived fungal peptides exerting anti-HSV properties are represented by aspergillipeptides D and E [74], simplicilliumtide J, verlamelines A and B [75], and acremonpeptides A and B, as well as Al(III)-acremonpeptide D [76] (Figure 4). Aspergillipeptides D and E were isolated from a fermentation broth of the marine-gorgonian-derived fungus *Aspergillus* sp. SCSIO 41501. The fungus was isolated from the gorgonian *Melitodes squamata* collected from the South China Sea. Aspergillipeptide D is a new cyclopentapeptide, while its counterpart aspergillipeptide E is a new linear peptide which was likely the direct product of a high concentration of l-tryptophan added to the medium. Based on a plaque-reduction assay, aspergillipeptides D and E had significant antiviral activity against HSV-1 (IC_50_ = 9.5 µM and 19.8 µM, respectively). Moreover, aspergillipeptide D also showed a remarkable antiviral effect against acyclovir-resistant clinical (ACV) isolates of HSV-1 (HSV-1-106 and HSV-1-135), with an IC_50_ value of about 12.5 µM [74]. Further study of its mechanism of antiviral action revealed that this cyclic pentapeptide seemed not to act on viral particle inactivation, attachment, or penetration in the early stages of infection, but rather on the inhibition of the viral glycoprotein gB. As the most highly conserved glycoprotein and a class III viral fusion protein, gB engages in the interaction and fusion of the virion and the host cell membrane in a direct manner. The study showed that aspergillipeptide D decreased the expression and synthesis of the gB1 protein and its localization in the endoplasmic and Golgi apparatus, leading to reduced viral intercellular spread. Moreover, in the presence of the peptide, the number of cellular proteins related to translation, ribosomal structure, biogenesis, posttranslational modification, protein turnover, chaperones, and the cytoskeleton—which interact with gB—were also extensively decreased. Therefore, these results highlight the great potential of aspergillipeptide D for HSV-1 infection therapy—particularly for ACV-resistant strains [115]—which is in accordance with the computational-peptidology-approach-based study by Glossman-Mitnik et al. [116]. Their very recent computational analysis of the chemical reactivity and bioactive properties corroborated the potential of this AVP through its interaction with different target receptors. Indeed, the in silico ADMET (Absorption, Distribution, Metabolism, Excretion, and Toxicity) characteristics of aspergillipeptide D showed the absence of toxicity as well as fine absorption and distribution properties [116].

Regarding simplicilliumtide J, this new cyclohexadepsipeptide, along with previously known analogues verlamelines A and B, has been isolated from the marine fungal strain *Simplicillium obclavatum* EIODSF 0210 collected from marine sediment in the depths of the East Indian Ocean. Simplicilliumtide J had a notable anti-HSV-1 activity, with an IC_50_ value of 14 µM, while the known analogues verlamelines A and B were first reported to possess antiviral activity against HSV-1, with IC_50_ values of 16.7 and 15.6 µM, respectively, based on a plaque assay. Based on the structure–activity relationship analysis, their potent antiviral activity could be attributed to the presence of the lactonized 5-hydroxytetradecanoic acid moiety in these cyclic hexapeptides [75]. However, the mechanism of antiviral activity of these cyclopeptides remains puzzling.

Additionally, with regard to marine fungal anti-HSV peptides, Luo et al. (2019) [76] also reported new antiviral cyclopeptide siderophores bearing hydroxamate isolated from the marine fungus *Acremonium persicinum* SCSIO 115, namely, acremonpeptides A and B, and the aluminum complex of acremonpeptide D, or Al(III)-acremonpeptide D. Interestingly, these cyclopeptides are defined by three 2-amino-5-(*N*-hydroxyacetamido)pentanoic acid (*N^5^*-hydroxy-*N^5^*-acetyl-l-ornithine) metal-ion-chelating moieties. They found that these peptides possessed antiviral activity against HSV-1, with EC_50_ values of 16, 8.7, and 14 µM, respectively. A further mode-of-action study revealed that these AVPs could inhibit viral DNA replication, as shown by the downregulated UL42 gene transcription in HSV.

In addition to sponges and tunicates as the richest sources of antiviral compounds from marine invertebrates [86,100,117,118], several compounds from mollusks have also been found to have antiviral activity, such as anti-HSV activity. One recent example of marine-mollusk-derived AVPs is myticin C. Myticin C was found in mussel hemolymph and hemocytes of the Mediterranean mussel *Mytilus galloprovincialis*. This cysteine-rich antimicrobial peptide initially showed antiviral activity against an aquatic pathogen fish rhabdovirus—ostreid herpesvirus 1 (OsHV-1) [119]. The antiviral activity of this peptide against OsHV-1 led to further investigation of antiviral effects against human HSVs, including HSV-1 and HSV-2. Interestingly, a modified synthetic myticin C with a Tat-derived cell-penetrating motif (Myt-Tat) and the nanoencapsulation peptide (with nonionic surfactant nanovesicles or nanosomes) showed antiviral inhibition of HSV-1 and HSV-2, as indicated by their SI values. The putative SI values for Myt-tat were >8.21 towards HSV-1 and >10.5 towards HSV-2, whereas for encapsulated myticin C they were >7.69 towards HSV-1 and >8.32 towards HSV-2. In addition, based on the virus plaque titers, the encapsulated peptides exerted a viral-inhibitory effect at 22.5 µM or above, implying thorough suppression of viral replication. The nanovesicles can fuse with the plasma membrane, delivering the peptide into the cytoplasm. The study suggested that myticin peptides selectively block an intracellular stage of the viral replication course, rather than direct viral inactivation or a virucidal effect. However, further investigation of the detailed mode of antiviral action of these peptides is still required [77].

Moreover, concerning marine-vertebrate-derived AVPs, the promiscuous peptide *Pa*-MAP (multiple active peptides from *Pleuronectes americanus*) was derived from the antifreeze peptide (AFP) HPLC-8, originally isolated from the skin of the polar fish *P. americanus* [120]. This synthetic alanine-rich peptide has shown antiviral activity against the viruses HSV-1 and HSV-2. Based on a titer-reduction assay, the peptide caused 82% HSV-1 inhibition (at 45 µM) and 90% HSV-2 inhibition (at 23 µM), while 94% and 97% inhibition of HSV-1 and HSV-2, respectively, were observed at the concentration of 90 µM. Thus, these results suggest that the hydrophobic residue in this peptide could facilitate interaction with the viral envelope and phospholipids at the viral surface for inhibiting viral infection [121]. Moreover, further investigation by Franco et al. [78] proved that *Pa*-MAP exerts a direct virucidal effect, preventing viral adsorption or entry, which supports the previous finding [121,122].

## 7. Anti-HCV (Hepatitis C Virus) Marine AVPs

**Figure 5:**
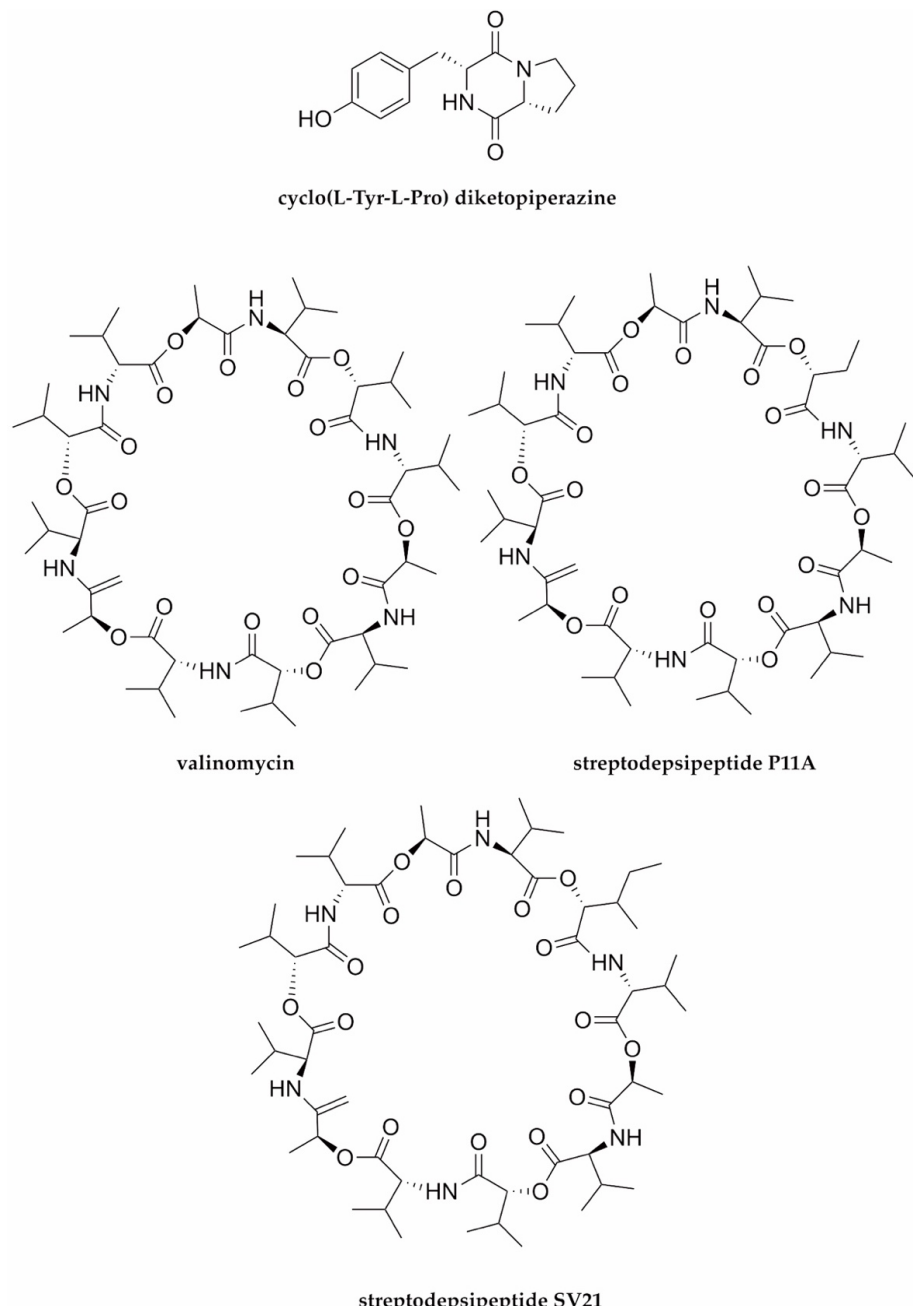
The chemical structures of marine-microbial-derived peptides possessing anti-HCV properties.

Viral hepatitis C, which leads to cirrhosis, carcinoma, and liver failure in humans, continues to be a serious global health burden. However, there is still no protective vaccine available for this disease. Moreover, despite its significant advancements, HCV therapy still faces the major issues of high cost and adverse effects. Thus, there is an urgent need to discover and develop potent anti-HCV drugs, including those derived from marine natural products. In recent years, some marine-microbial-derived peptides have been reported to have promising anti-HCV properties, including cyclo(l-Tyr-l-Pro) diketopiperazine [79], valinomycin. and its analogues streptodepsipeptide P11A and streptodepsipeptide SV21 [80] (Figure 5 and Table 1).

Cyclo(l-Tyr- l-Pro) is a cyclic dipeptide diketopiperazine isolated from the Red Sea black sponge *Spongia officinalis*-associated fungus *Aspergillus versicolor*. This cyclodipeptide diketopiperazine exhibited potent activity against HCV NS3-4A protease, with an IC_50_ value of 8.2 µg mL^−1^ [79]. This would thus suggest that this compound targets viral replication.

Along with the known valinomycin and its analogue streptodepsipeptide P11A, another new analogue—cyclodepsipeptide streptodepsipeptide SV21—was very recently isolated from a sea-cucumber-associated bacterium *Streptomyces* sp. SV21, collected from Lampung, Indonesia [80]. Indeed, the antiviral activity of valinomycin has been recently reported to inhibit the coronavirus SARS-CoV-2 [123]; however, these current reported antivirals valinomycin and its derivatives demonstrated very significant inhibition of infectivity against HCV compared with the positive control epigallocatechin gallate (about 0–5% infectivity vs. 20% infectivity) [80]. Epigallocatechin gallate has been regarded as a strong HCV inhibitor, blocking early viral entry and attachment to the cell surface [124]. However, the mode of anti-HCV activity of these compounds has not been evaluated, although it has been speculated that valinomycin interferes with the binding of the coronavirus glycoprotein to the host cells [125]. However, it has been suggested that the symmetry of the ring system is likely vital for the bioactivity of valinomycin and those analogues, based on the structure–activity relationship analysis [80].

## 8. Anti-SARS-CoV-2 Marine AVPs

**Figure 6:**
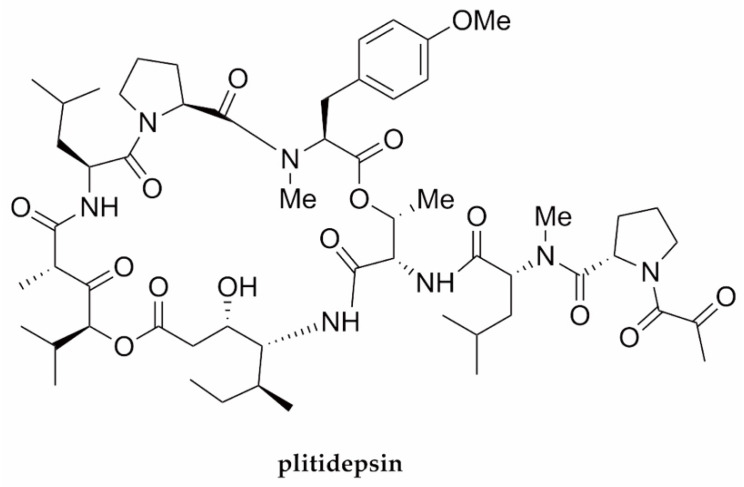
The chemical structure of plitidepsin—a marine-derived peptide exhibiting anti-SARS-CoV-2 activity.

Several literature reviews have been extensively compiled in an attempt to search for potential antimicrobial peptides against SARS-CoV-2 [28,61,62,67,126,127]. Moreover, drug repurposing to accelerate the identification of available drugs that can be used to treat coronavirus disease 2019 (COVID-19) has also been broadly discussed [128,129,130,131,132,133,134,135]. However, a recent marine AVP with potential anti-SARS-CoV-2 activity is exemplified by plitidepsin (Figure 6 and Table 1), which is known under the trademark Aplidin^®^ (PharmaMar, S.A., Madrid, Spain) for the treatment of multiple myeloma [136,137,138].

Plitidepsin is a cyclodepsipeptide originally isolated from the Mediterranean marine tunicate *Aplidium albicans*. This cyclodepsipeptide can now be obtained via total synthesis [139]. Computationally aided in silico studies of plitidepsin by molecular docking and molecular dynamic simulation related to its protease-inhibitory activity have shown its great binding effectiveness with the main protease of SARS-CoV-2 compared with other available drugs, e.g., hydroxychloroquine, remdesivir, and lopinavir. Thus, this peptide has shown tremendous potential for drug repurposing against COVID-19 [140,141]. Moreover, very recently, White et al. (2021) [142] verified that plitidepsin exhibited anti-SARS-CoV-2 activity, with an IC_90_ value of 0.88 nM, which was stronger than remdesivir in the human cell line hACE2-293T by a factor of 27.5, with limited toxicity. By employing a drug-resistant mutant (a mutated eukaryotic translation elongation factor 1A or eEF1A in 293T cells), they also demonstrated that the antiviral activity of plitidepsin was assisted by the prohibition of the known target eEF1A, which is a vital host factor for viral replication. In addition, it has been demonstrated that plitidepsin treatment could reduce the replication of SARS-CoV-2 by two orders of magnitude and diminish lung inflammation in vivo in murine models. Hence, these gathered findings show that plitidepsin is a promising therapeutic candidate for COVID-19. In addition to successful assessment through a phase I/II clinical trial by PharmaMar, it is now undergoing phase II/III trials for the treatment of COVID-19 [137,142]. Results from in vitro investigations also demonstrate that the peptide has a potent antiviral function against all variants, with viral load reduction of 99% in animal lung tissue. Moreover, positive effects in phase I and II clinical trials on patients hospitalized with COVID-19 were also recorded [143]. In addition, Guisado-Vasco et al. have also recently reported that plitidepsin succeeded in providing the cure for prolonged viral SARS-CoV-2 replication in a patient with hematological malignancy and depleted B cells [144].

## 9. Concluding Remarks and Future Perspective

In summary, marine organisms such as sponges, tunicates, mollusks, microbes, and fish have recently produced potent peptides with moderate-to-significant antiviral activity in vitro. AVPs obtained or reported from these marine sources during the period covered in this short review, from 2011 to 2021, comprise about 25 compounds. Most of them are fungal-sourced natural peptides, and are mainly derived from non-ribosomal peptides. Conversely, ribosomal divamide A, myticin C, and *Pa*-MAP are (semi-)synthetic variants whose origins are amenable for structural modifications. These recently discovered marine cationic peptides act against the enveloped viruses via different mechanisms. Some AVPs have been known to be virucidal, act on viral entry, bind to the viral envelope, or inhibit viral replication and intercellular spread, while the antiviral activity of some others remains elusive.

Interestingly, it has been markedly shown that almost all of these recently reported promising peptides are cyclic peptides, ranging from cyclic dipeptide diketopiperazine to cyclodepsipeptides. Due to its reduced conformational flexibility, the cyclic feature has been suggested to facilitate increased cell permeability and better bioactivity compared to those with a linear structure. Indeed, the cyclopeptide aspergillipeptide D was found to be more active than the linear peptide aspergillipeptide E. Cyclopeptides are also more resistant to degradative proteases due to their lack of C- and N-termini, and are endowed with entropic (Gibbs free energy) benefits in terms of molecular binding and recognition [145,146,147]. Therefore, these favorable properties make this class of peptides a captivating candidate for therapeutics. Improved therapeutic targeting can be attained through structural changes, including cyclization, the use of stable d-amino acids, and chemical modification. However, critical issues remain in the development of cyclopeptides for therapeutics, including bioavailability and cell permeability. Innovative methods could overcome these challenges, such as the application of cell-penetrating peptides and nanoencapsulation [146,147,148,149], as applied in the antiviral peptide myticin C (myt-Tat) against HSV-1 and HSV-2 [77].

Although several marine peptides are currently being subjected to preclinical and clinical investigations, or have even hit the market [22], none of them are antiviral lead compounds or drugs yet. In vivo animal evaluation or therapeutic application have not yet been reported for almost all of the aforementioned promising AMPs with antiviral activity. However, through drug repurposing to cope with COVID-19, the synthetic anticancer drug plitidepsin has demonstrated anti-SARS-CoV-2 effects in vitro and in vivo, and is now undergoing phase II/III clinical trials. With the advancement in (meta)genomic and synthetic biology approaches, marine-peptide-based antiviral drug discovery and development will be accelerated. Fermentation technology—especially for microbial-metabolite-based peptides—can replace chemical synthesis for their scalable production. Furthermore, given their promising antiviral activities, the potential of AVPs with possibly reduced toxicity and fewer adverse effects in preclinical and clinical treatments should be further explored, aided by virtual screening via computational peptidology [150]. Apparently, marine peptides will play a pivotal role in developing lead compounds and drugs for various viral infectious diseases in the near future.

## Figures and Tables

**Table 1:** Recent reported antiviral bioactive peptides derived from marine organisms during 2011–2021.

Targeted Virus	Peptide	Biosynthetic Class	Origin	IC_50_/EC_50_/SI/Infectivity	Mechanism of Antiviral Action (Target of Inhibition)	Reference
HIV-1	Mirabamides E–H	Cyclodepsipeptides/NRPs	Sponge *Stelletta clavosa*	121, 62, 68, 41 nM	Viral fusion	[68]
HIV-1	Stellettapeptines A and B	Cyclodepsipeptides/NRPs	Sponge *Stelletta* sp.	23 and 27 nM	Viral entry (Viral membrane)	[69]
HIV-1	Mollamide F	Cyclodepsipeptide/NRP	Tunicate *Didemnum molle* PNG07-2-050	78 μM (cytoprotective) 39 μM (HIV-integrase)	Viral integrase	[70]
HIV-1	Malformin C	Cyclopeptide/NRP	Endophytic fungus *Aspergillus niger* SCSIO Jscw6F30	1.4 μM	ND *	[71]
HIV-1	Divamide A	Lanthipeptide/ribosomal peptide	Tunicate *Didemnum molle* E11-036	0.225 μM	PE binding	[72]
H1N1/H3N2	Asperterrestide A	Cyclopeptide/NRP	Endophytic fungus *Aspergillus terreus*	20.2 and 0.41 μM	ND *	[73]
HSV-1	Aspergillipeptide D	Cyclopeptide/NRP	Endophytic fungus *Aspergillus* sp. SCSIO 41501	9.5 μM (HSV-1) 12.5 M (ACV-HSV-1)	Viral intercellular spread (Viral glycoprotein gB)	[74]
HSV-1	Aspergillipeptide E	Linear peptide/NRP	Endophytic fungus *Aspergillus* sp. SCSIO 41501	19.8 μM	ND *	[74]
HSV-1	Simplicilliumtide J	Cyclodepsipeptide/NRP	Fungus *Simplicillium obclavatum* EIODSF 0210	14.1 μM	ND *	[75]
HSV-1	Verlamelines A and B	Cyclodepsipeptide/NRPs	Fungus *Simplicillium obclavatum* EIODSF 0210	16.7 and 15.6 μM	ND *	[75]
HSV-1	Acremonpeptides A and B	Cyclopeptide/NRPs	Fungus *Acremonium persicinum* SCSIO 115	16 and 8.7 μM	Viral replication	[76]
	Al(III)-acremonpeptide D	Cyclopeptide/NRPs	Fungus *Acremonium persicinum* SCSIO 115	14 μM	Viral replication	[76]
HSV-1/HSV-2	Myticin C	Ribosomal peptide	Mollusk *Mytilus galloprovincialis*	7.69–8.21/8.32–10.5	The intracellular phase of viral replication	[77]
HSV-1/HSV-2	*Pa*-MAP	Ribosomal peptide	polar fish *Pleuronectes americanus*	82% (45 μM)/90% (23 μM)	Virucidal effect	[78]
HCV	Cyclo(l-Tyr-l-Pro) diketopiperazine	Cyclopeptide diketopiperazine/NRP	Endophytic fungus *Aspergillus versicolor*	8.2 μg mL^−1^	NS3-4A protease	[79]
HCV	Valinomycin; streptodepsipeptides P11A and SV21	Cyclodepsipeptides/NRPs	Bacterial symbiont *Streptomyces* sp. SV21	0–5%	ND *	[80]
SARS-CoV-2	Plitidepsin	Cyclodepsipeptide/NRP	Tunicate *Aplidium albicans*	0.88 nM	Viral replication (eEF1A)	[80]

* ND = not yet described.

## Data Availability

Not applicable.
